# Handlebar hernia–unusual complication from blunt trauma

**DOI:** 10.1093/jscr/rjac195

**Published:** 2022-06-03

**Authors:** Bruno Vieira, Ana Melo, Carina Gomes, Leandro Lajut, Artur Ribeiro, Urânia Fernandes, Gonçalo Guidi, Clara Leal, João Pinto-de-Sousa

**Affiliations:** Department of Surgery, Centro Hospitalar Trás-os-Montes e Alto Douro, Vila Real, Portugal; Department of Surgery, Centro Hospitalar Trás-os-Montes e Alto Douro, Vila Real, Portugal; Department of Surgery, Centro Hospitalar Trás-os-Montes e Alto Douro, Vila Real, Portugal; Department of Surgery, Centro Hospitalar Trás-os-Montes e Alto Douro, Vila Real, Portugal; Department of Surgery, Centro Hospitalar Trás-os-Montes e Alto Douro, Vila Real, Portugal; Department of Surgery, Centro Hospitalar Trás-os-Montes e Alto Douro, Vila Real, Portugal; Department of Surgery, Centro Hospitalar Trás-os-Montes e Alto Douro, Vila Real, Portugal; Department of Surgery, Centro Hospitalar Trás-os-Montes e Alto Douro, Vila Real, Portugal; Department of Surgery, Centro Hospitalar Trás-os-Montes e Alto Douro, Vila Real, Portugal

## Abstract

Handlebar hernia is a rare entity, mainly resulting from blunt abdominal trauma with a sudden deceleration mechanism. Diagnosis of handlebar at admission may be difficult because the rupture of abdominal wall layers often is not clinically recognized in the emergency department, which requires a high degree of suspicion to identify theses lesions. It is very important to rule out the presence of intra-abdominal injuries, and in adults, surgical repair is needed. Herein, the case of an adult man who presented to the emergency department with blunt abdominal trauma caused by a motorcycle handlebar is described.

## INTRODUCTION

Traumatic injury is the leading cause of death in people younger than the age of 44 [1]. Abdominal trauma is a major determinant of morbidity and mortality in young individuals worldwide [2, 3]. The blunt impact of the handlebar into the abdomen from a bicycle, a motorcycle or a seat belt can result in significant underlying trauma with minimal external visible signs of injury [4]. Although possible and troublesome, the frequency of associated visceral injury within these hernias seems to be low [5–7]. According to the literature most reported cases are managed with surgical exploration and simple suture repair [8]. Nevertheless, despite minimal signs on clinical examination, the involved mechanism and history should raise the suspicion of significant underlying muscular disruption, and the eventual presence of intra-abdominal lesions.

## CASE REPORT

**Figure 1:**
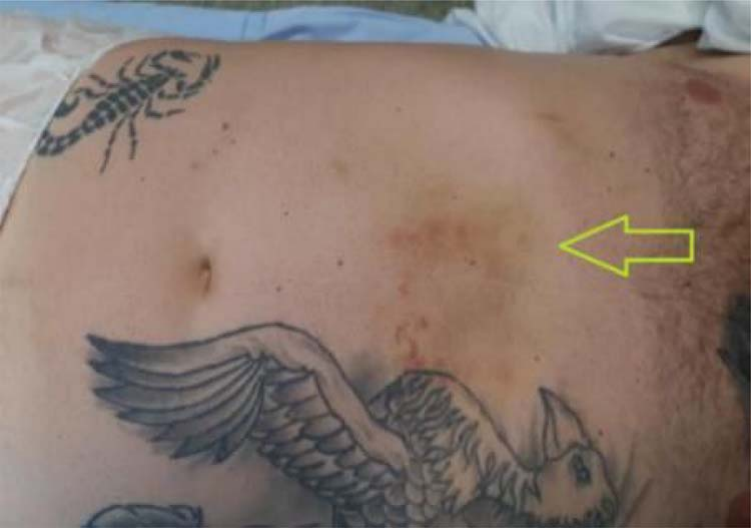
Handlebar sign—bruising in the epigastric region (green arrow). Patient lying supine.

**Figure 2:**
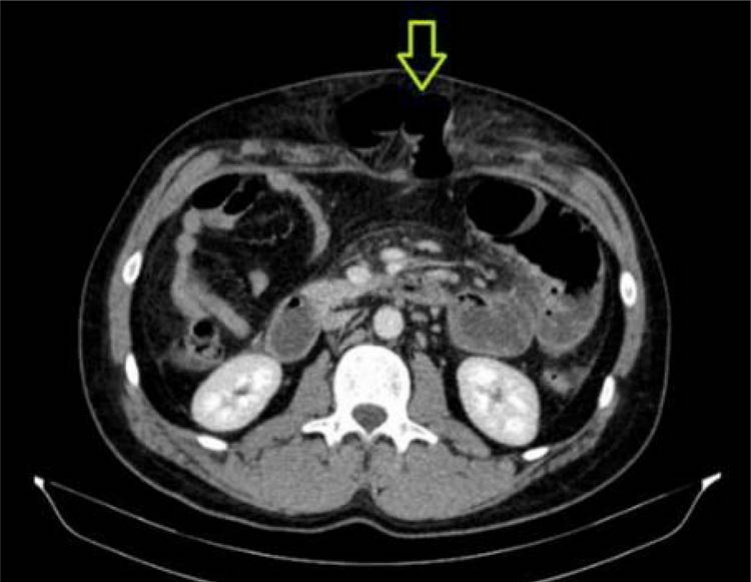
CT abdomen showing abdominal wall hernia containing a loop of small intestine (green arrow).

**Figure 3:**
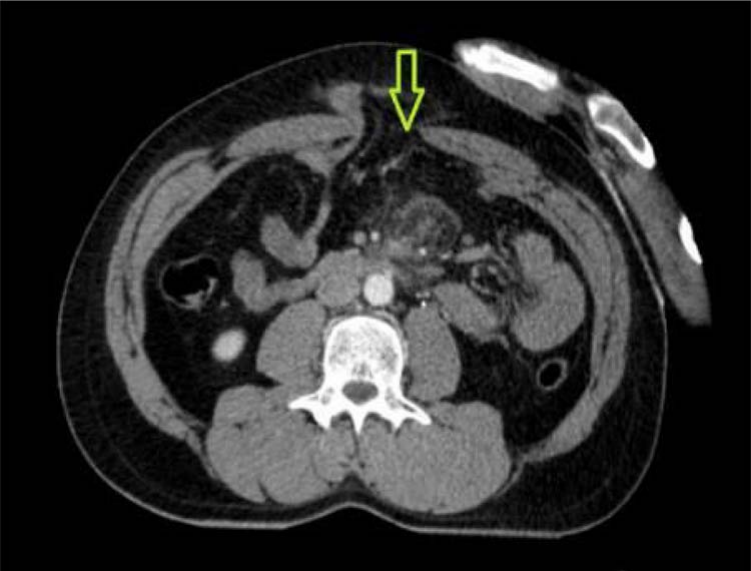
CT abdomen showing abdominal wall defect (green arrow).

**Figure 4:**
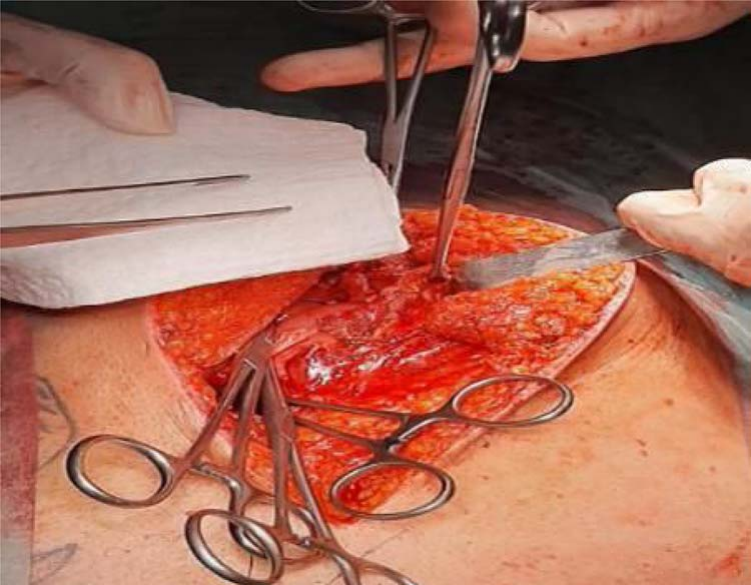
Operative findings. Disruption to muscular layers and peritoneum from handlebar injury, revealing small bowel. Clips placed on posterior rectus sheath.

**Figure 5:**
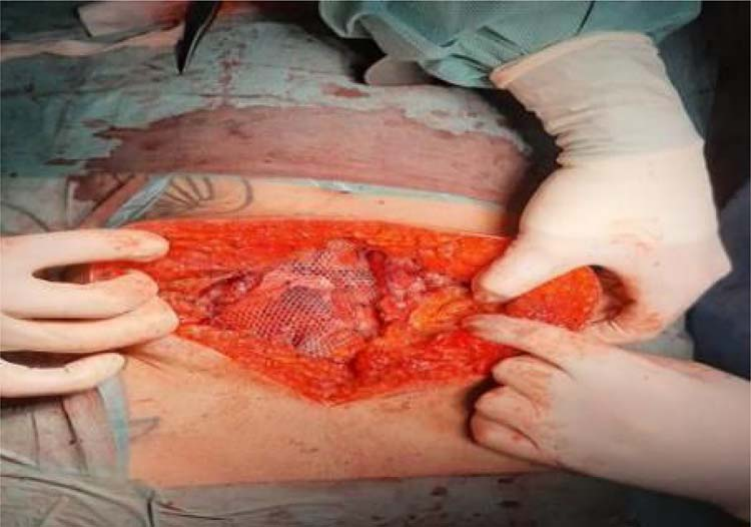
Operative findings. Retromuscular sublay mesh repair.

A 41-year-old man presented to the emergency room soon after falling from his motorcycle and hitting his handlebars in the epigastric region. The patient’s vital signs and initial laboratory studies were normal. Physical examination showed a soft tissue bulge in the epigastric region with superficial ecchymosis and tenderness to palpation. The swelling was obvious on standing and less evident when lying supine (Fig. 1). Focused Assessment with Sonography for Trauma (FAST) performed in the emergency room did not show free fluid in the abdominal cavity. Computed tomography (CT) scan showed intestinal loops protruding through a defect in the abdominal wall into the subcutaneous space (Figs 2 and 3). Therefore, a surgical approach for handlebar hernia treatment was decided on the first day of hospitalization. A defect throughout the entire abdominal wall, including the fascia, muscular layers and peritoneum, with bowel protruding into the subcutaneous space, was observed during surgery (Fig. 4). There was no blood or fecal contamination in the area immediately surrounding the lesion, and exploration of the bowel loops did not reveal signs of intra-abdominal injury. Based on this, hernia repair was performed with prosthetic material (Fig. 5). As there was no evidence of intra-abdominal injury, local wound exploration provided the best anatomic layered repair with subsequent minimal residual defect and improved long-term cosmesis. The defect was repaired in layers, and the patient postoperative course was uneventful.

## DISCUSSION

Handlebar hernia is an example of traumatic abdominal wall hernia, which are defined as a herniation through disrupted muscles and fascia, without skin penetration and with no previous defect on the local of injury [9]. Despite its rarity, handlebar hernias should be suspected when significant blunt force is applied to the abdominal wall from a handlebar injury. In some cases, physical examination reveals abdominal wall tenderness and ecchymosis [9], as was the case here reported, but there may no obvious signs on physical examination. Thus, the diagnosis can be easily missed, or limited to that of a local hematoma [10]. Sometimes the Valsalva maneuver can give additional help in the differential diagnosis between hematoma and traumatic hernia [11]. Therefore, a high level of suspicious must be remembered due to the involve mechanism and kinetic energy. As consequence imaging methods, namely ultrasound and CT scan, are very important to achieve a correct diagnosis [1, 12–14].

Handlebar hernias are often under type I abdominal wall hernias according to Wood *et al.* proposed classification [15], and thus associated intra-abdominal injuries are rare. Although handlebar injury was the reason for our case, the presentation was typical of type III hernia. Indeed, the energy from the accident of a high-speed motorcycle may explain the extent of injury in our patient. However, even the high energy involved in the accident, the patient of this case suffered no intra-abdominal besides the abdominal wall rupture, which agrees with other reports [8, 16], but contrasts to other reports in which incarceration and eventually intra-abdominal ischemic lesions are present [5, 17–20], mainly in situations of such high energy involved.

In pediatric ages, as well as in selected adult cases, handlebar hernias can often successfully be managed conservatively [17, 21–23], but in most of adult cases, given the high kinetic energy involved, as was the case reported, the extent of the default usually mandates surgical correction. Definitive treatment includes surgical exploration with primary repair of all tissue layers of the abdominal wall. The choice between tissue repair or mesh repair is dependent on the extent of the abdominal wall defect as well as on the presence of other intra-abdominal lesions, namely bowel perforation and peritonitis [17, 24].

Summing up, in cases of traumatic abdominal wall defects, a high level of clinical suspicion is needed to rule out the diagnosis of traumatic abdominal wall hernia, strongly considering the involved mechanism. Although not common the presence of intra-abdominal lesions may be considered on the patient management.

## CONFLICT OF INTEREST STATEMENT

The authors have no conflicts of interest to declare.

## FUNDING

The authors declare that no financial support was received.

## ETHICS STATEMENT

This manuscript is in accordance with the rules of our Institutional Ethics Committee.
